# Supplementary material for: Lived experiences and coping strategies of people living with Glaucoma in Nigeria: A qualitative study
Source: PLoS One. 2025 Jun 16;20(6):e0325258. doi: 10.1371/journal.pone.0325258 (PMC12169591; doi:10.1371/journal.pone.0325258)
Supplement: Attachment — Submitted filename: Response to Reviewers.docx [file pone.0325258.s002.docx]

**Response to reviewers’ comments**

**Dear Editor,**

We appreciate the comments from the reviewers which has strengthened our study. Below, we have provided point by point responses to each of the comments. In the manuscript, the revised sections are highlighted in red fonts.

**Reviewer #1**

Thank you for the opportunity to read the paper on the experiences and coping strategies of people living with glaucoma in Nigeria. The paper is important and brings forth the experiences of individuals with glaucoma. Here are a few suggestions to improve the paper:

1. *Does the fact that the interviews were conducted with a group of patients from outpatient clinics in large hospitals affect the interviewees' group?*

**Response:** No. The recruitment of participants from this large hospital has no influence on interviewees group. Participants in qualitative research are not recruited to be representative of the population. This is because unlike quantitative studies, qualitative studies do not seek to generalize their findings (Levitt, 2021). The study explored the experiences of the participants concerning the subject under investigation.

*Is there a population group that was not included in the interviews?*

No. The targeted population was included. The study utilized a purposive sampling method to recruit its participants. According to Cambell et al. (2020), purposive sampling, a common technique in qualitative research is a non-probabilistic sampling method that involves deliberately selecting participants based on their relevance to the population characteristics and the study's objectives.

2. *The first theme in the paper deals with psychological response. However, there are references to issues whose connection to psychological response is not clear, such as the ability of buying medications. Is the fear the psychological symptom? It was not sufficiently clear.*

**Response:** Thanks for the comment. It appears that the reviewer may have misunderstood the study. The first theme deals with “psychosocial response” and not “psychological response.” Psychosocial responses are the emotional, cognitive, behavioural, and social responses that participants experienced in response to their glaucoma diagnosis. As seen in theme 1, there were emotional reactions of anxiety, fear and depression. Participants expressed concerns regarding the possibility of vision loss, leading to significant anxiety. For instance, the fear of spending their limited finance on medications also made them anxious. A participant also became depressed and wanted to commit suicide by drinking bleach. The fear of becoming a burden or the stigma associated with vision impairment also made some participants isolate themselves, withdrawing from social interactions. No change was made.

3. *In the discussion, you raised the issue of the difficulties faced by glaucoma patients in several aspects and the lack of knowledge people have about the disease. Difficulty due to illness is natural, as is a lack of knowledge about the disease. However, and this is the main weakness of the paper, there is not enough discussion on possible ways to mitigate these difficulties. It is important to note how you believe the state or society in Nigeria can address the challenges raised in the study, whether from a social, economic, or medical perspective.*

**Response:** Thanks for this comment. The discussion on possible ways to mitigate these difficulties have been included in the recommendation section of the manuscript. See lines 677-684 which reads like so:

The government at different levels in Nigeria can mitigate the challenges faced by people with glaucoma by improving access to affordable eye care services, especially in rural communities by establishing more eye care clinics and training additional ophthalmologists and optometrists. In addition, public awareness campaigns can be conducted to educate Nigerians about glaucoma, and the financial burden can be lessened by subsidizing the cost of medication and services, offering grants, or creating health insurance schemes. Furthermore, the government can liaise with local and international funding organizations to secure funding and resources for glaucoma research, treatment, and awareness programs.

4. *Regarding the economic issue - the economic difficulty was mentioned both in the results and in the discussion, but I did not fully understand the problem. How much does monthly glaucoma treatment cost in Nigeria? Is there public funding for this treatment? What are the amounts involved, and how does this impact the individual patient?*

**Response:** Thanks for this comment. We have addressed this in lines 595 to 605.

Glaucoma patients experience economic difficulty due to direct and indirect costs. The direct medical cost include cost associated with regular consultations for disease monitoring, h, investigations, treatment, and surgery. The indirect non-medical costs includes decreased productivity and employment opportunities, strained family finances, caregiver expenses, transportation, government purchase programs, guide dogs, and nursing home care (Varma et al. 2011). In Nigeria, majority of glaucoma patients are not subscribed to the National Health Insurance Scheme, and they fund their treatment out of pocket (Bello et al., 2023). The average monthly direct cost of glaucoma management in Nigeria was NGN 9,954 (about $6); and glaucoma patients can spend over one-tenth of their monthly income on glaucoma care alone (Bello et al., 2023).

**Reviewer #2**

Lived experiences and coping strategies of people living with Glaucoma in

Nigeria -A qualitative study. Many thanks for the opportunity to review this interesting study. Using the COREQ Checklist I have identified several areas where this submission could be improved which I hope you will find helpful.

Introduction:

P1 Line 90 *– fixing is highlighted as one of the activity limitations – can more clarification be provided here – fixing what? Some readers may be unfamiliar with this term in relation to domestic tasks.*

Response: Thanks for the comment. We have replaced the word “Fixing” with “repairs”

P2 Line 115 *missing word - critical factor in healthcare [13], is also a key benefit with exposure to narratives and experiences the*

Response: This has been revised and now read:

Hope, a crucial element in healthcare [13], is also a significant benefit gained from exposure to the narratives and experiences of others in similar circumstances [14].

*P 4 Line 117 – *I understand that there has been little research in Nigeria on this topic, can you draw on research conducted in other areas for your background section?*

**Response:** This has been discussed in the introduction. The relevant section in Lines 92 -110 now reads:

Few studies have examined the experiences of individuals living with glaucoma in Nigeria (Kyari et al., 2016; Abdull et al., 2016; Mbadugha & Onakoya, 2014). These studies have primarily focused on understanding the disease, access to care, delayed presentation for care, and the socioeconomic impact of living with glaucoma. Other studies have highlighted low glaucoma awareness, challenges in accessing care that lead to delayed presentation, and subsequent socioeconomic deprivation (Obasuyi et al., 2024; Okoye et al., 2018; Kyari et al., 2016; Onyia et al., 2022; Abdull et al., 2016; Mbadugha & Onakoya, 2014). The studies identified several factors contributing to limited access to glaucoma care, including individual-level factors such as inadequate education, unemployment, gender, rural residence, the cost of care, and religious beliefs (Obasuyi et al., 2024; Okoye et al., 2018). In addition, institutional-level factors, such as inadequate equipment and manpower shortages, significantly limit access to care, often leading patients to engage in risky help-seeking behaviours (Obasuyi et al., 2024; Okoye et al., 2018). Consequently, traditional healers have become the initial point of consultation for individuals experiencing reduced vision or those who do not notice improvement with treatment (Abdull et al., 2016).

Methods:

*I would suggest that this is a qualitative study with the sample taken from a larger cross-sectional study (which is not apparent until the methods) as there is no inclusion of any references or methods relating to a phenomenological approach therefore I suggest removing reference to this study being a phenomenological study as highlighted in the abstract.*

**Response:** Done. We have removed the reference to phenomenological study.

*Line 132 conducted on adults – conducted with adults might be more appropriate terminology.*

**Response:** Done. This has been revised to “conducted with”

Line 135 *A subsample of phase 1 participants were then invited to join the second phase of the study comprising of semi-structured, in-depth, face-to-face interviews covering the experiences and coping strategies of these participants – can you provide more information about how were this sample selected and recruited? Did you contact all 100 participants with glaucoma participating in the larger study? How were participants approached? Did any participants drop out?*

**Response:** Thanks for the comment. Although this was included in the article, we have made it clearer considering the questions you raised. The current section addressing these concerns now reads:

The study was conducted in two phases. In Phase 1, participants completed relevant questionnaires using validated measuring scales, and in Phase 2, the interviews were done. In the first phase of the study, survey respondents were given preliminary information about the second phase, which involved qualitative interviews. At the end of the survey, they were asked if they would be interested in participating in an interview and could indicate their interest by ticking a box in the questionnaire. Participants were then asked to provide their preferred contact details. All individuals who expressed interest and provided their contact information were reached by phone to confirm their eligibility. A total of eighteen potential participants expressed interest, and all were allowed to take part if eligible. In all, twelve (12) participants were recruited and provided with the participant information statement and consent form. A purposive sampling approach was employed to intentionally select participants based on their relevance to the population characteristics and the study's objectives.

*Line 148 participants attended the clinic during the study period, - please provide the study period*

**Response:** The study period has been provided.

……..attending the eye clinics Calabar, Nigeria, from 10 March 2022 and 3 June 2022.

Line 171 – *Participants could rearrange the protocol if they wished - do you mean change the order of the questions asked? Where was the location of the interviews conducted? Was anyone else present? Were any participants interviewed a second time or was all data captured with one interview each?*

**Response**: These details have been made clearer in the relevant section in the methods. The section now reads like this:

Participants were provided with the interview itinerary in advance but were given the option to choose the venue and time for the interviews. However, all participants preferred to have the interviews scheduled and conducted in the Resource Room of the Department of Ophthalmology at the University of Calabar Teaching Hospital, led by one of the researchers (KEA). The interviews were comprehensive, and all data was captured in a single session per participant, with no follow-up interviews conducted. Participants were interviewed by one of the researchers (KEA.), who was trained and experienced in using the qualitative interview schedule.

Line 174 - *Pre-interview guide was used and probes employed during the interview to elicit further details and enhance meaning, as required – does this include the interview questions? Has this been provided as a supplementary file? Was this piloted prior to use? Did the researcher make field notes during the interview?*

**Response**: These have been addressed.

The interview guide, which included the interview questions, was pre-tested with a cohort of individuals with high myopia attending the eye clinic to assess its suitability. However, this cohort was excluded from the final data collection. Probes were employed during the actual interviews to elicit further details and enhance the depth of responses as needed. Interviews lasted for an average time of 55 minutes and were audio-recorded using a digital recorder. The interview guide has been provided as a supplementary file. Additionally, the researcher took field notes during the interviews to capture contextual details and observations.

Line 189 - *The researchers agreed that for a topic to become a theme at least two participants would have made substantive comments on the issue during their interviews. – does this method align with the Braun & Clarke approach to thematic analysis? What about the subsequent stages of the thematic analysis – how did the researchers refine and define themes? Was data saturation reached? Were transcripts or analysis returned to participants for checking? Did you use any software to assist with data analysis?*

**Response:** The data analysis section has been revised to provide clarity on how the thematic analysis aligned with the Braun & Clarke approach, addressed the refinement of themes, explained the absence of member checking and software, and the researchers' perspective on data saturation.the relevant section inf the manuscript now reads like so:

Audio files were transcribed verbatim by one of the co-authors (K.E.A.), and transcripts were de-identified to maintain confidentiality (participant quotes are referred to in this paper using codes). Only members of the research team had access to the transcripts. Thematic analysis was conducted using the six-step approach outlined by Braun and Clarke [21]. To ensure data familiarization, K.E.A. transcribed the interviews verbatim. Both K.E.A. and co-author (N.M.), who are experienced qualitative researchers, thoroughly read the transcripts multiple times, line by line, to gain a deep understanding of each participant’s personal story and immerse themselves in the data.

During the initial coding process, the researchers agreed that for a topic to be identified as a theme, at least two participants must have made substantive comments on the issue during their interviews. Following this, the initial themes were refined and reviewed through an iterative process to enhance their clarity, coherence, and accuracy in conveying the overall meaning [21]. Finally, the themes were defined and named using descriptive labels that captured the core ideas they represented. Member checking was not performed, as the researchers felt that transcribing the interviews verbatim ensured an accurate representation of the participants' views. Given the diverse range of participant experiences, data saturation was not a primary consideration for this study. Data analysis was conducted manually without the use of software.

Line 193 – *Thank you for your section on Catalytic validity and reflexivity – could you expand further to highlight if the researcher conducting the interviews already knew the participants?*

**Response:** This has been clarified. The relevant section now reads:

The researchers were aware that prior familiarity with participants might undermine the credibility of research findings (Johnson et al., 2020). As a result, the researcher who conducted the interview was not familiar with any of the participants.

Results:

*Line 423 is repetition from line 389 – please revise your text.*

**Response:** Lines 422-426 are the summary of Theme 2: Impact on everyday life

Discussion:

*Line 580 beginning with Hence doesn’t read very well can you please reword.*

**Response:** “Hence” has been removed and replaced with: “For this reason, the findings may….”, or “As a result the findings may….”
