# Supplementary material for: Lived experiences and coping strategies of people living with Glaucoma in Nigeria: A qualitative study
Source: PLoS One. 2025 Jun 16;20(6):e0325258. doi: 10.1371/journal.pone.0325258 (PMC12169591; doi:10.1371/journal.pone.0325258)
Supplement: Attachment — Submitted filename: Response to the Reviewer 4-5-2025.docx [file pone.0325258.s004.docx]

Response to the Reviewer

Dear Editor,

Thank you for the opportunity to revise and resubmit our manuscript, ‘Lived Experiences and Coping Strategies of People Living with Glaucoma in Nigeria—A Qualitative Study.’ We are grateful to the reviewer for providing valuable feedback that will improve our paper.

We have revised the manuscript to the reviewer’s comments. The table below shows our response to each comment. As advised by the Editor, all modifications in the manuscript are highlighted using track changes.

We trust that the revisions have reasonably addressed the reviewer’s concerns and that our manuscript is now suitable for publication in PLOS ONE.

Once again, thank you for the opportunity to revise and resubmit our manuscript.

Kind regards,

Dr Kelechukwu Ahaiwe

|  | **Reviewer’s Comment** | **Author’s Response** | **Author’s Action** |
| --- | --- | --- | --- |
| 1 | **Materials and Methods:** 1. This was a cross-sectional study conducted with adults living with vision impairment due to glaucoma, high myopia, diabetic retinopathy and low vision a. It is important that the methodology and research paradigm of this work is stated clearly. As is, the opening lines gives the impression that this is a quantitative approach to the RQ proposed by the authors. b. It may seem that this is part of a larger work, however, this paper focussed majorly on Glaucoma. The authors may need to focus their work on Glaucoma. | Thank you for the feedback and suggestions provided in your review. This study is part of a larger project that utilized a mixed-methods approach to explore various aspects of visual impairment, including diabetic retinopathy, high myopia, and low vision. However, this paper focuses exclusively on participants diagnosed with glaucoma and is entirely a qualitative study, employing a phenomenological approach. As such, the analysis, results, and discussion focus specifically on the themes derived from the experiences of participants living with glaucoma. The manuscript has been updated to reflect this focus in the introduction and methods sections. | The manuscript has been updated to reflect this focus in the introduction and methods sections. |
|  | 2. In Phase 1, participants completed relevant questionnaires using validated measuring scales a. What were these scales? Please state these scales and include references. b. How did these scales answer the research objectives of this paper? c. Were these scales validated in the population being studied before use? | In the first phase of the study, participants completed validated questionnaires to capture baseline information on their vision-related quality of life and psychosocial well-being. The following scales were used:   1. World Health Organization Quality of Life Scale (WHOQOL-BREF) 2. The Beck Depression Inventory (BDI) scale   However, these validated measuring scales were utilized for the quantitative study aspect of the larger study and provided contextual understanding of the participants' functional limitations and emotional states, which enhanced the qualitative study in Phase 2. It is not utilized as the primary focus of this manuscript's data analysis. However, the questionnaires guided the researchers in understanding participants' experiences and informing the selection of the semi-structured interview questions during the detailed interviews. Given this, the scale did not contribute to the study's qualitative part, nor the discussion and results sections of the manuscript. Therefore, the description of the validated scale has been expunged from the manuscript | ‘Validated scales’ have been expunged from the manuscript since they were not used to collect qualitative data for this study. |
|  | 3. A total of eighteen potential participants expressed interest, and all were allowed to take part if eligible. In all, twelve (12) participants were recruited and provided with the participant information statement and consent form. a. Could the authors state why there was a 30% attrition rate in response to phase 2? | Like in every qualitative research, our aim in this study was not to generalize findings to a larger population. Instead, we sought to understand experiences, perceptions, and social processes in-depth. Unlike quantitative studies, qualitative studies do not adhere to a predetermined number of participants. Instead, qualitative studies focus on achieving data saturation—the point at which no new themes or insights are being revealed from the data. In this study, saturation was achieved with the 12 participants who took part in the interviews. The definition of saturation has been included in the manuscript to improve clarity.  This was not an attrition rate, actually, but the proportion that consented for interviewed after responding to the quantitative arm of the study | Lines 51, 140-148  The saturation definition below has been included in the manuscript and further explanation given to improve clarity. |
|  | 3. Purposive sampling facilitates better matching of the sample to the aims and objectives of this research, enhancing the integrity of research a. How was purposive sampling instrumental to achieving the aims of the study when   i. There were more females than males ii. The participants were Christians iii. The participants were well educated b. Even though this was highlighted as a limitation, was there a better approach? Could the authors have been more specific in their recruiting process, seeing as qualitative studies allow for such sampling methods? | Thanks for this observation. This study employed purposive sampling to recruit participants diagnosed with glaucoma who possessed at least one year of treatment experience. This approach ensured that participants provided rich information and deep reflective insights that aligned with the study's objectives.  While this method facilitated alignment with the research goals, it was skewed towards females, Christians, and individuals with tertiary education, and these characteristics have been acknowledged as limitations within the manuscript.  Nevertheless, considering the study's exploratory nature and the context of urban hospital-based recruitment, the sample accurately represented the demographic engaging with glaucoma services in the study setting. A more targeted recruitment strategy—incorporating outreach through community eye clinics, religious organizations, and support groups for individuals with low literacy—might have resulted in a more representative population. Therefore, future research could focus on diversity and benefit from utilizing maximum variation sampling to ensure a broader representation of educational, religious, and gender backgrounds. Moreover, a definition of the concept has been included to the manuscript to further explain the selection process. | A definition of the concept has been included to the manuscript to further explain the selection process:  recruiting the sample for focus groups and in-depth interviews. *Qualitative health research*, *11*(1), 117-126. |
| **2** | **RESULTS:** 1. Most participants disclosed that they felt sad and despondent on receiving the news of their glaucoma diagnosis. a. At what stage did these patients receive their diagnosis? Was this explored? i. The authors stated that they discussed with patients who had advanced glaucoma ii. The inclusion criteria was that the participants would have been receiving Glaucoma care for a year. The above points presuppose that some participants may have been diagnosed for longer than a year. If this is the case, exploring how they coped with the evolution of their disease provides more context to the discussion of the themes. | Thank you for this thoughtful comment. As stated, our inclusion criteria required participants to have been receiving glaucoma care for at least one year. However, we did not stratify participants based on the specific number of years since diagnosis. We acknowledge that the length of time since diagnosis can influence a patient’s lived experience and coping mechanisms, and this is an important area for further exploration. Accordingly, we have now included this point as a limitation in the revised manuscript and suggested it as a direction for future studies.  It is also worth noting that although participants may have varied in the duration since diagnosis, this variable did not emerge as a distinct theme in the data. Most participants consistently reported experiencing sadness and despondency upon initial diagnosis, regardless of the time since they were diagnosed. This suggests a shared emotional response to the diagnosis itself, which was more prominent in their narratives than the evolving nature of their condition over time. | Limitation revised to include this statement for future studies [lines 621-627] |
| **3** | **DISCUSSION** 1. In Nigeria, the majority of glaucoma patients are not covered by the National Health Insurance Scheme and must pay for their treatment out of pocket [33]. The average monthly direct cost of managing glaucoma in Nigeria is NGN 9,954 (approximately $6), meaning patients may spend over one-tenth of their monthly income on glaucoma care alone. a. The cost of glaucoma in Nigeria needs to be contextualised to provide clarity and understanding viz: i. Minimum wage ii. Regionally. See Omoti AE, Edema OT, Akpe BA, Musa P. Cost Analysis of Medical versus Surgical Management of Glaucoma in Nigeria. J Ophthalmic Vis Res. 2010 Oct;5(4):232-9. | Thanks for this suggestion. As requested, glaucoma management has been contextualised to reflect cost and regional implications in the discussion. The suggested reference was also added | Lines 560-584 |

Reference

Adio, A. O., & Onua, A. A. (2012). Economic burden of glaucoma in Rivers State, Nigeria. *Clinical Ophthalmology*, 2023-2031.

African Development Bank Group. 2019. Nigeria Policy Options for Accelerated Economic Transformation and Job Creation. Accessed 27 April 2025. <https://www.afdb.org/sites/default/files/documents/projects-and-operations/policy_options_for_accelerated_economic_transformation_and_job_creation_-_nigeria_country_diagnostic_note.pdf>

Aglipay, M., Wylie, J. L., & Jolly, A. M. (2015). Health research among hard-to-reach people: six degrees of sampling. *CMAJ*, *187*(15), 1145-1149.

Atiye, D. E., & Madu, I. T. U. (2024). Trade Unionism and the Politics of Minimum Wage Implementation in Bayelsa State, Nigeria, 2012-2022. *Journal of Global Economics and Business January*, *5*(16), 30-55.

Blukacz, A., Cabieses, B., Obach, A., Calderón, A. C., Álvarez, M. I., Madrid, P., & Rada, I. (2023). Promoting the participation of “hard-to-reach” migrant populations in qualitative public health research during the COVID-19 pandemic in Chile. *International journal of environmental research and public health*, *20*(3), 1956.

Delgado, M. F., Abdelrahman, A. M., Terahi, M., Miro Quesada Woll, J. J., Gil-Carrasco, F., Cook, C., ... & Gomes, J. A. (2019). Management of glaucoma in developing countries: challenges and opportunities for improvement. *ClinicoEconomics and Outcomes Research*, 591-604.

Ezeudu, T. S., & Tukur, B. (2024). Examining the Effects of High Poverty and Unemployment on Rural Urban Migration in Nigeria and its Consequences on Urban Resources and Rural Decline.

Kenealy, T. W., Hao’uli, S., & Arroll, B. (2015). A qualitative study of recruiting for investigations in primary care: plan, pay, minimise intermediaries and keep it simple. *SAGE Open Medicine*, *3*, 2050312115596649.

MacDougall, C., & Fudge, E. (2001). Planning and recruiting the sample for focus groups and in-depth interviews. *Qualitative health research*, *11*(1), 117-126.

National Bureau of Statistics. 2019. Poverty and inequality in Nigeria 2019: Executive Summary. Accessed 27 April 2025. <https://www.nigerianstat.gov.ng/elibrary/read/1092>

Omoti, A. E., Edema, O. T., Akpe, B. A., & Musa, P. (2010). Cost analysis of medical versus surgical management of glaucoma in Nigeria. *Journal of ophthalmic & vision research*, *5*(4), 232.

Saunders, B., Sim, J., Kingstone, T., Baker, S., Waterfield, J., Bartlam, B., ... & Jinks, C. (2018). Saturation in qualitative research: exploring its conceptualization and operationalization. *Quality & quantity*, *52*, 1893-1907.

Sebele-Mpofu, F. Y. (2020). Saturation controversy in qualitative research: Complexities and underlying assumptions. A literature review. *Cogent Social Sciences*, *6*(1), 1838706.

Yang, L., QI, L., & Zhang, B. (2022). Concepts and evaluation of saturation in qualitative research. *Advances in Psychological Science*, *30*(3), 511.
